# Supplementary material for: Spatial Landscape Structure Influences Cross-Species Transmission in a Rabies-like Virus Model
Source: Microorganisms. 2025 Feb 14;13(2):416. doi: 10.3390/microorganisms13020416 (PMC11858330; doi:10.3390/microorganisms13020416)
Supplement: Supplementary file 1 [file microorganisms-13-00416-s001.zip › Supplementary figures S1-S17.pdf]

SUPPLEMENTARY MATERIAL

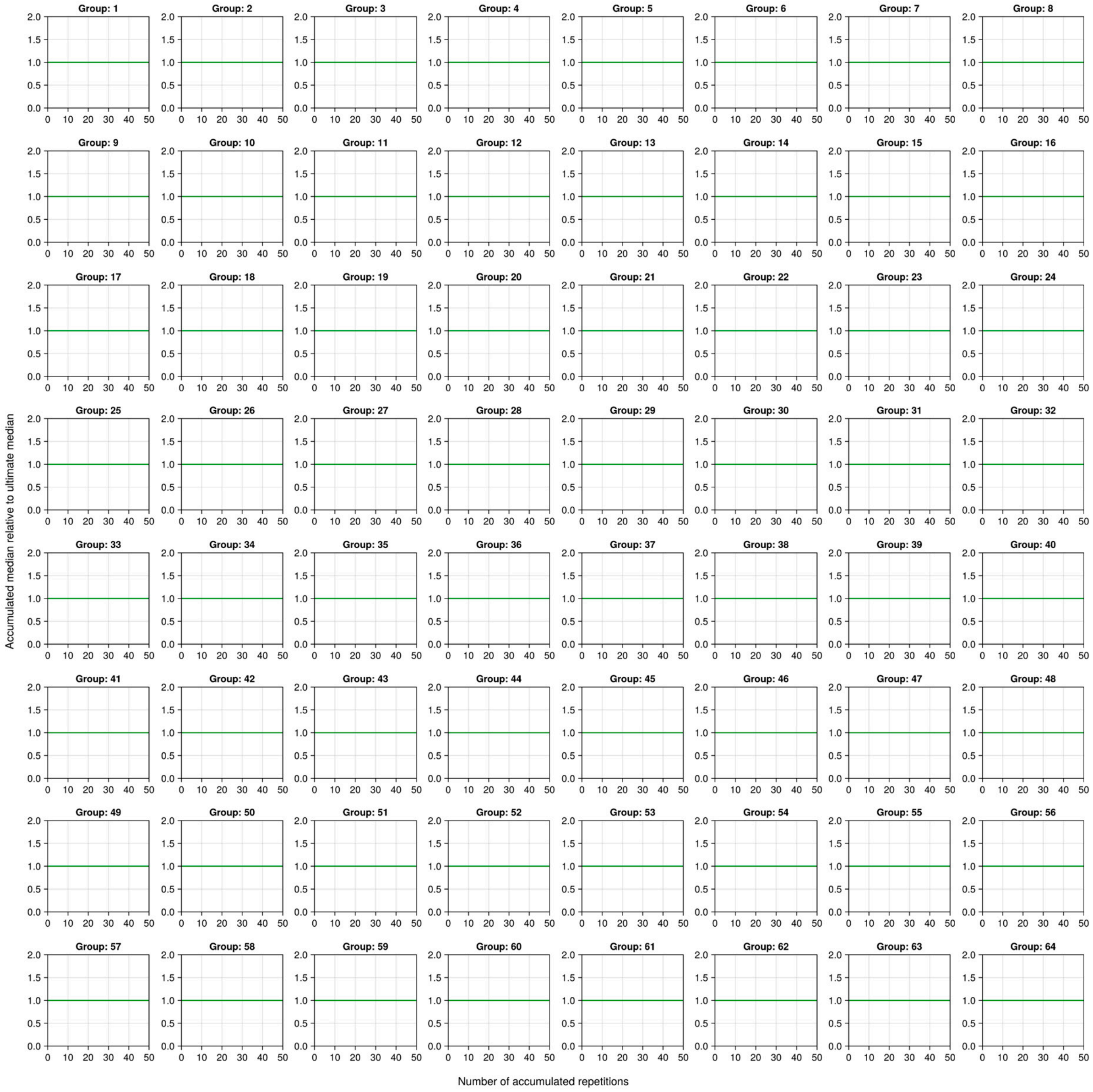

Figure S1. The cumulated median-to-ultimate median ratio for each simulation replicated in the baseline homogeneous model. Each dot represents the ratio for an individual replicate simulation. Only the first 64 of the 640 landscapes are shown for illustrative purposes.

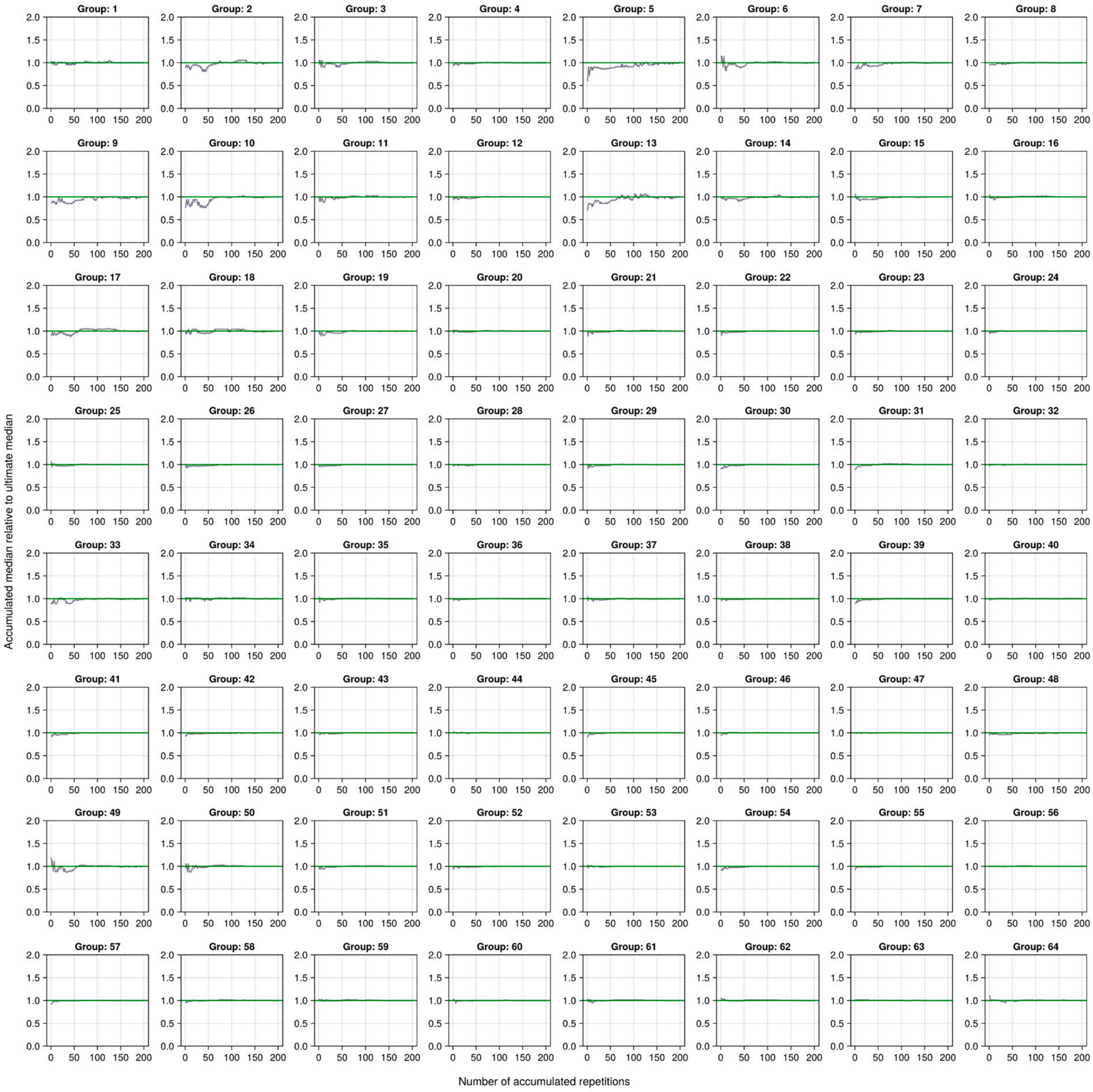

Figure S2. The cumulated median-to-ultimate median ratio for each simulation replicated in the baseline heterogeneous model. Each dot represents the ratio for an individual replicate simulation. Only the first 64 of the 640 landscapes are shown for illustrative purposes.

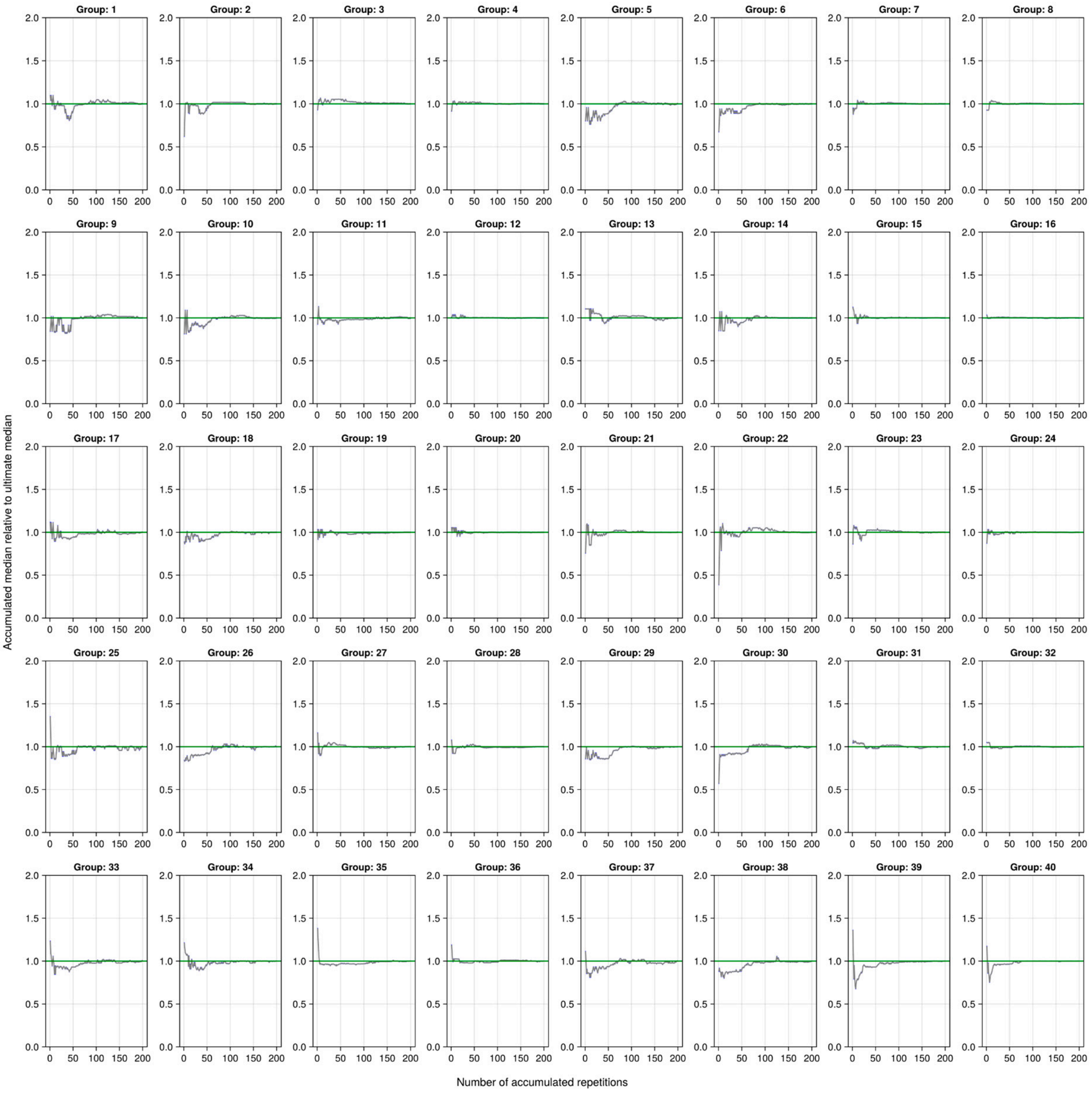

Figure S3. Cumulated median-to-ultimate median ratio for each simulation replicated in the species-specific heterogeneous model. Each dot represents the ratio for an individual replicate simulation. Only the first 40 of the 400 landscapes are shown for illustrative purposes.

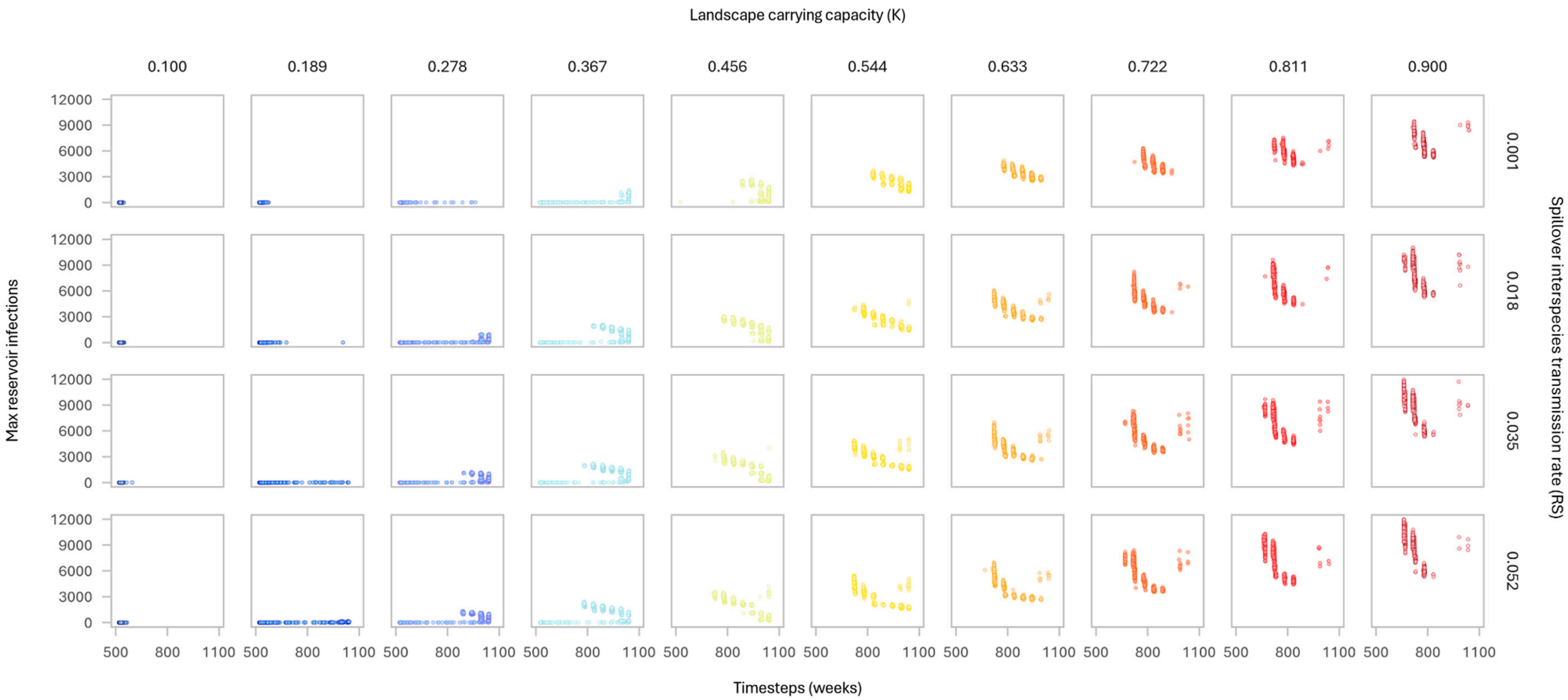

Figure S4. Timesteps (in weeks) at which the maximum number of reservoir cases occurs, plotted against landscape carrying capacity (K) and spillover interspecies transmission rate (RS). Each dot represents a replicate simulation.

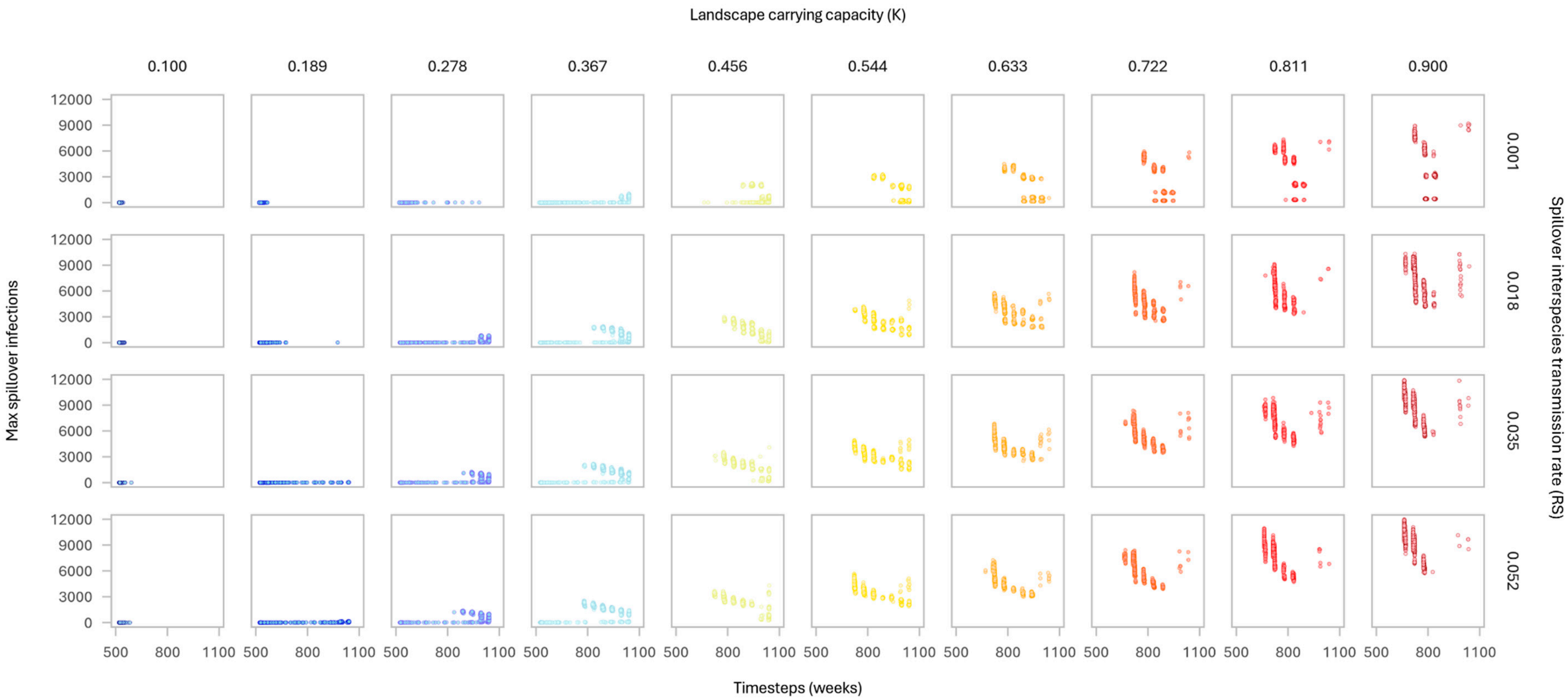

Figure S5. Timesteps (in weeks) at which the maximum number of spillover cases occurs, plotted against landscape carrying capacity (K) and spillover interspecies transmission rate (RS). Each dot represents a replicate simulation.

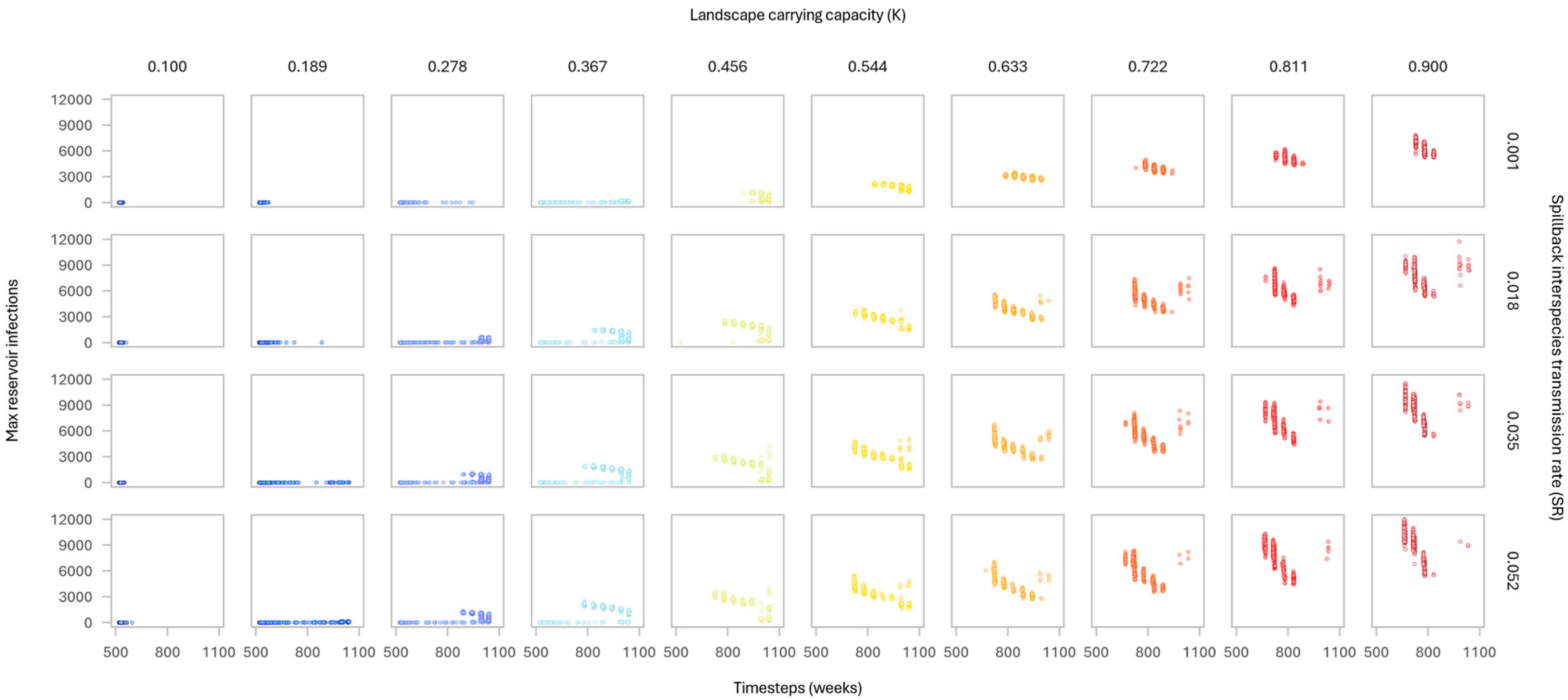

Figure S6. Timesteps (in weeks) at which the maximum number of reservoir cases occurs, plotted against landscape carrying capacity (K) and spillback interspecies transmission rate (SR). Each dot represents a replicate simulation

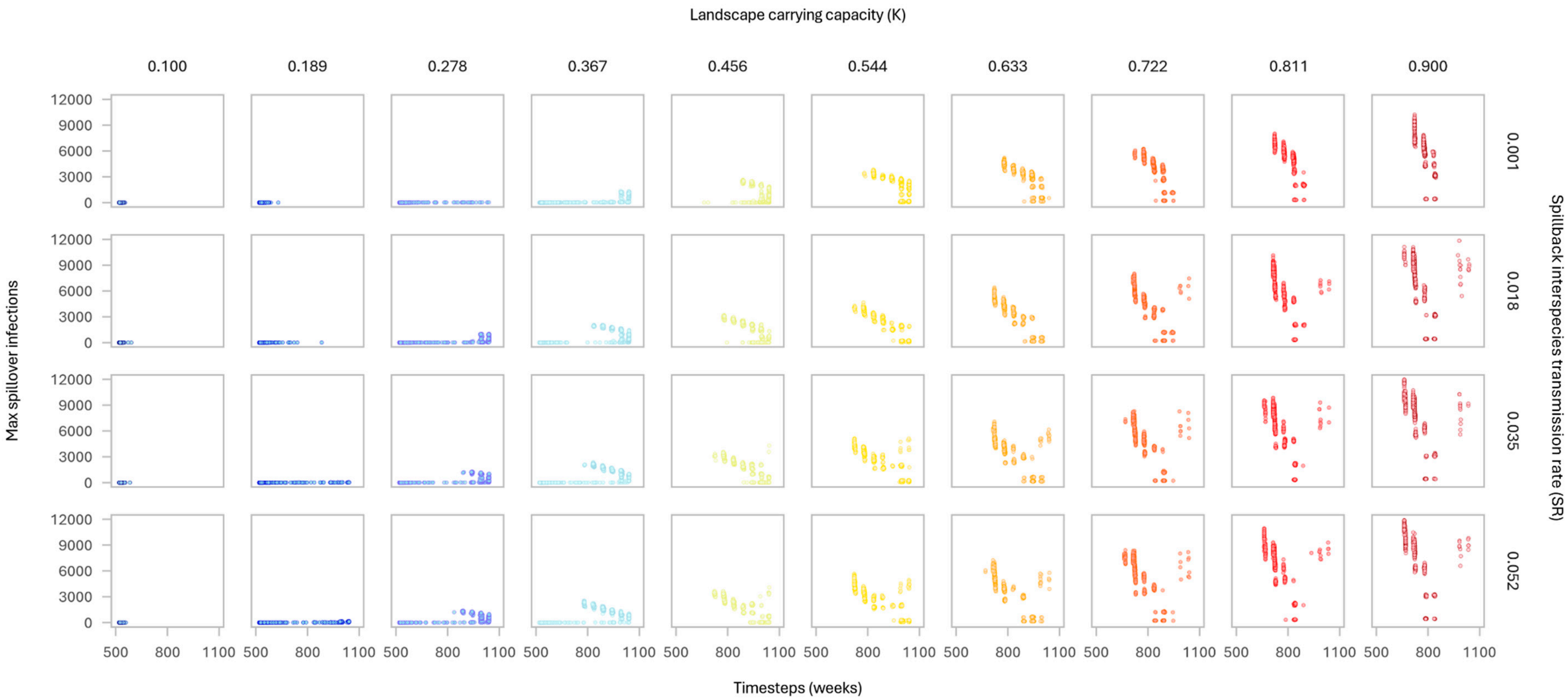

Figure S7. Timesteps (in weeks) at which the maximum number of spillover cases occurs, plotted against landscape carrying capacity (K) and spillback interspecies transmission rate (SR). Each dot represents a replicate simulation.

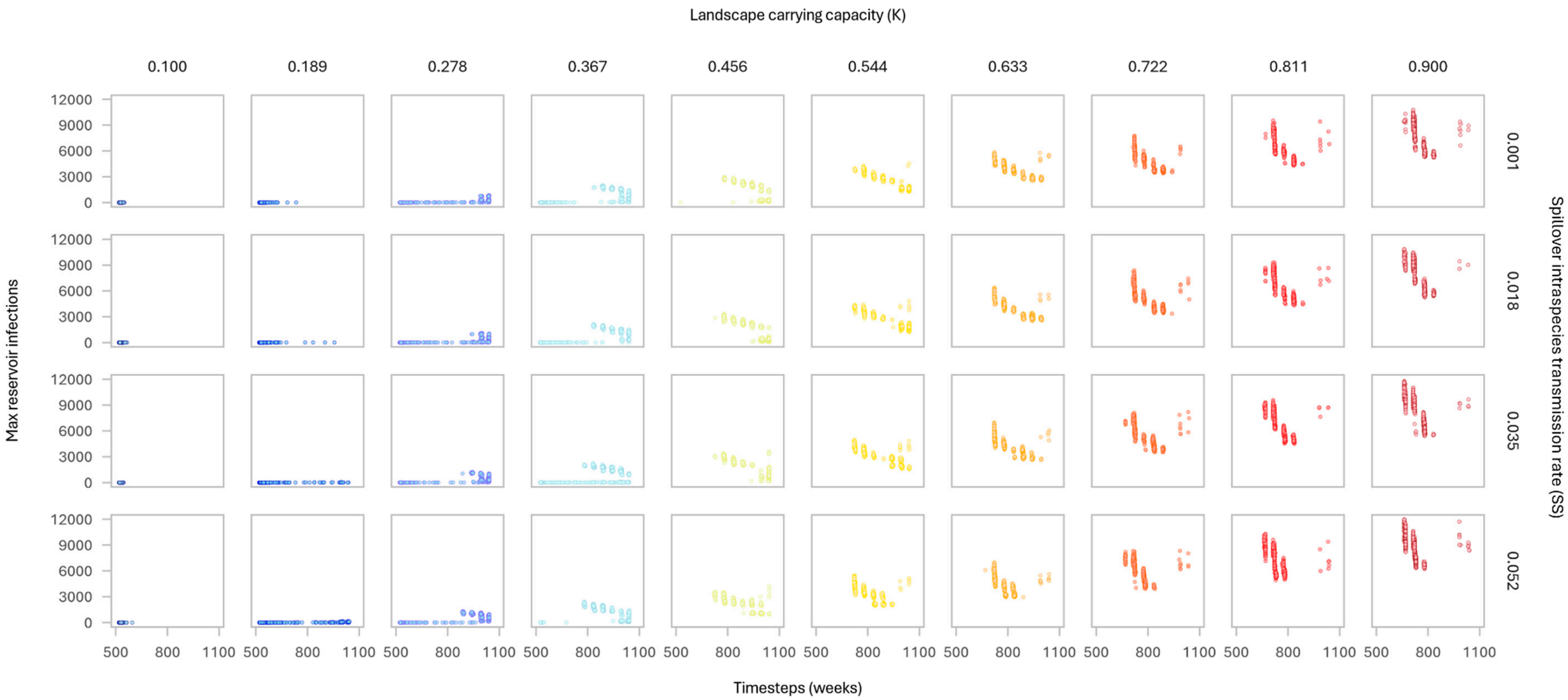

Figure S8. Timesteps (in weeks) at which the maximum number of reservoir cases occurs, plotted against landscape carrying capacity (K) and spillback intraspecies transmission rate (SS). Each dot represents a replicate simulation.

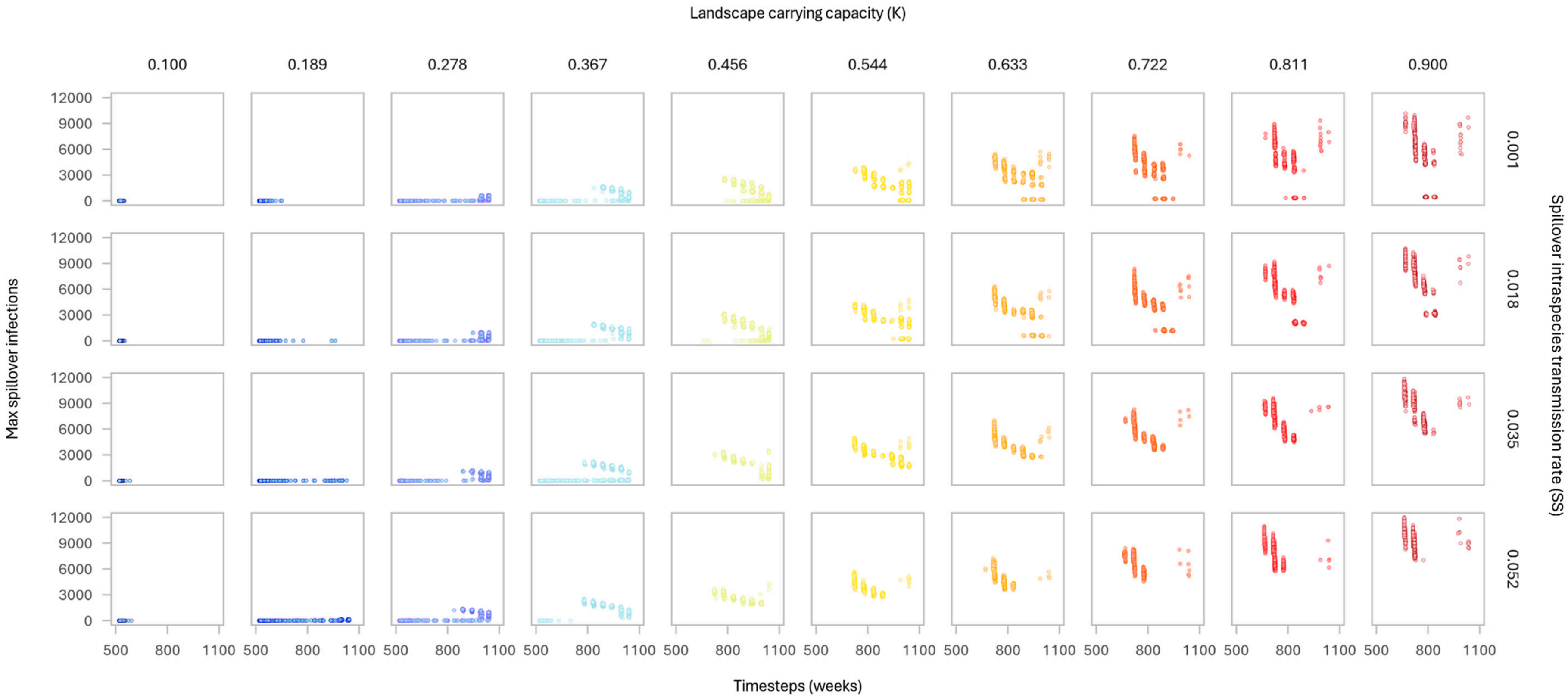

Figure S9. Timesteps (in weeks) at which the maximum number of spillover cases occurs, plotted against landscape carrying capacity (K) and spillback intraspecies transmission rate (SS). Each dot represents a replicate simulation.

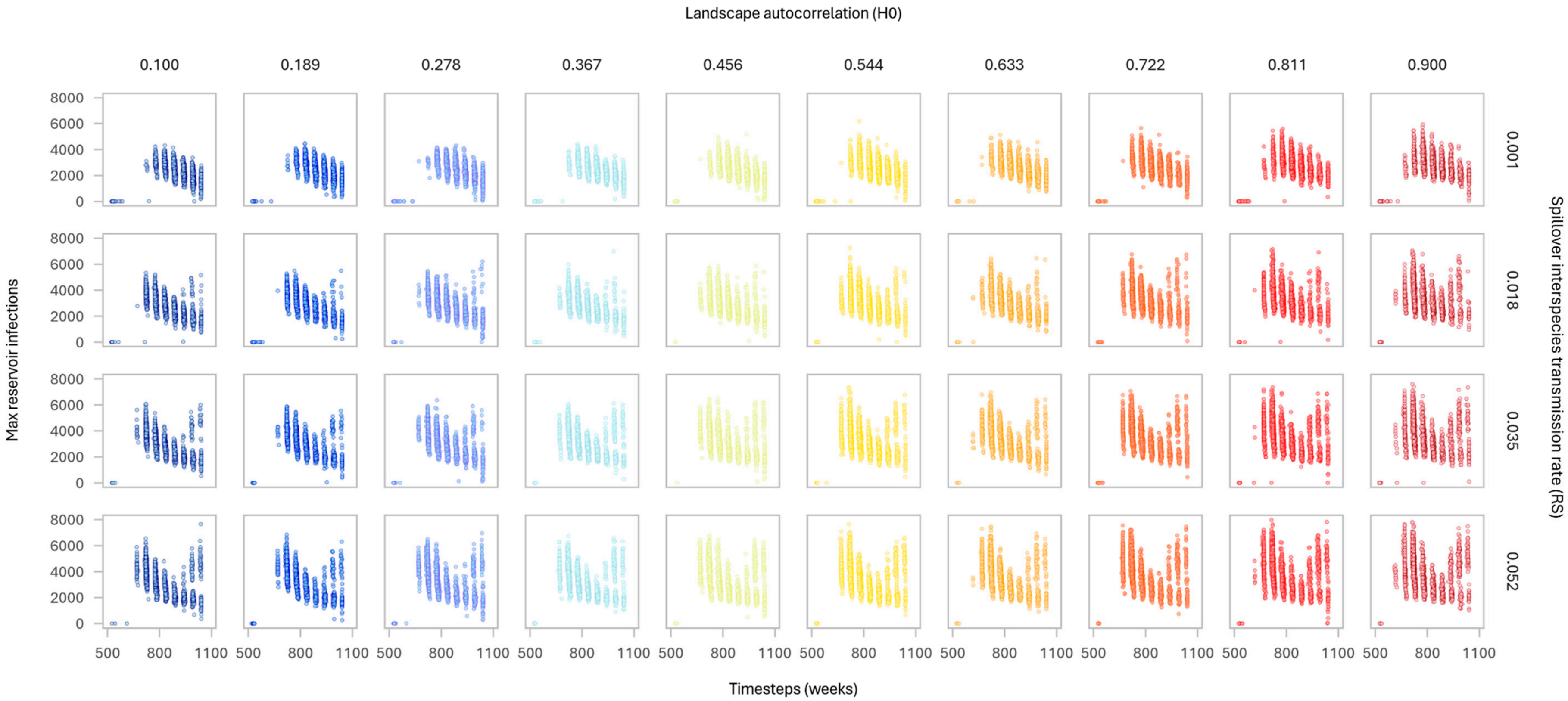

Figure S10. Timesteps (in weeks) at which the maximum number of reservoir cases occurs, plotted against landscape autocorrelation (H_0) and spillover interspecies transmission rate (RS). Each dot represents a replicate simulation.

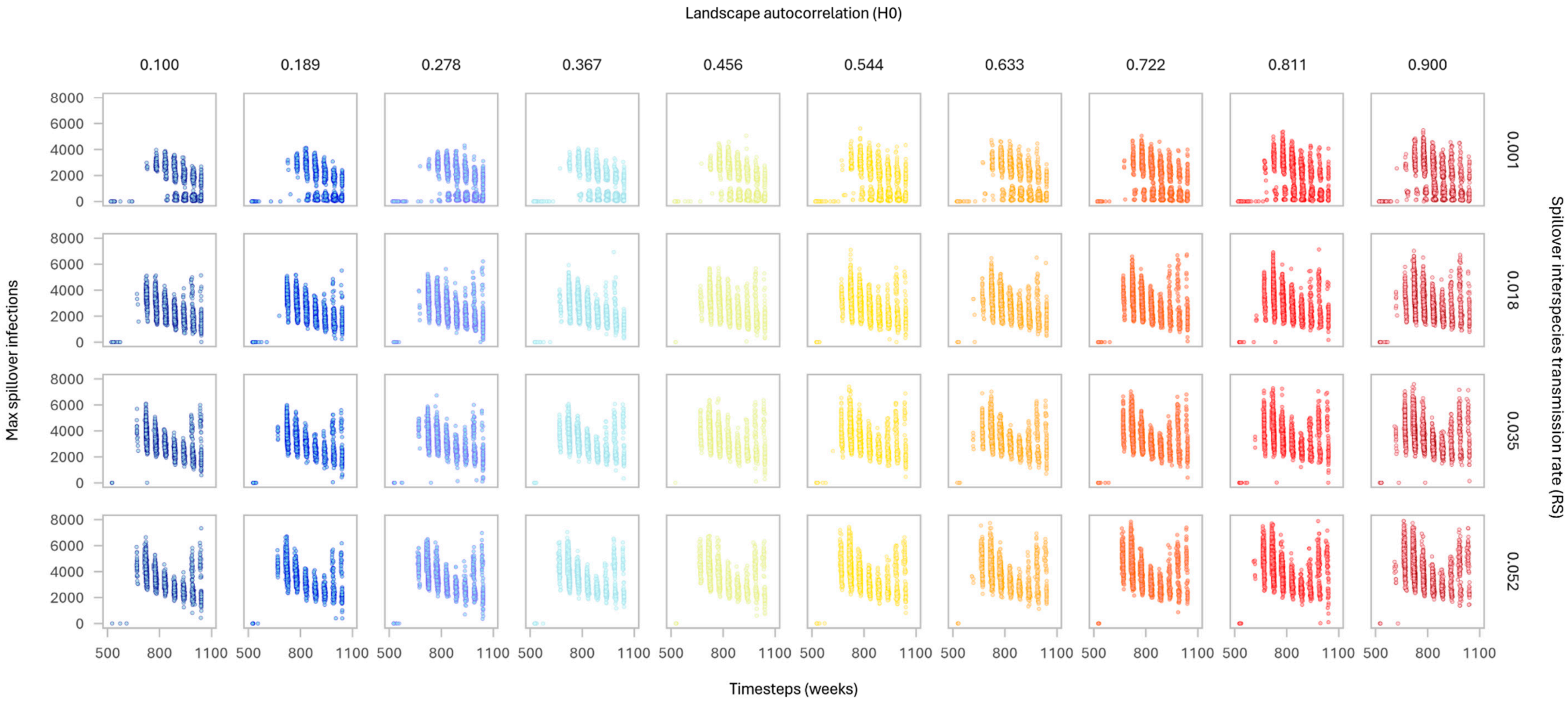

Figure S11. Timesteps (in weeks) at which the maximum number of spillover cases occurs, plotted against landscape autocorrelation (H_0) and spillover interspecies transmission rate (RS). Each dot represents a replicate simulation.

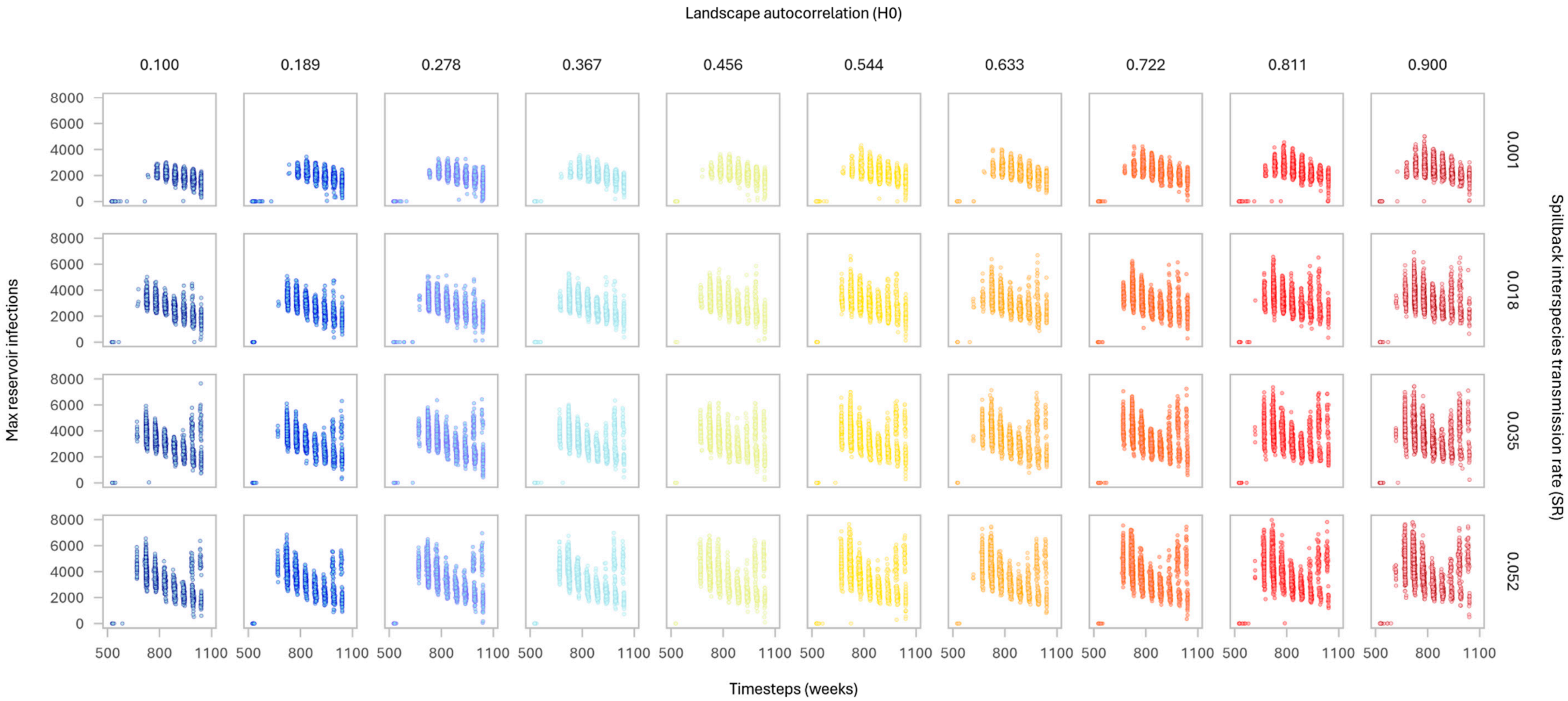

Figure S12. Timesteps (in weeks) at which the maximum number of reservoir cases occurs, plotted against landscape autocorrelation (H_0) and spillback interspecies transmission rate (SR). Each dot represents a replicate simulation.

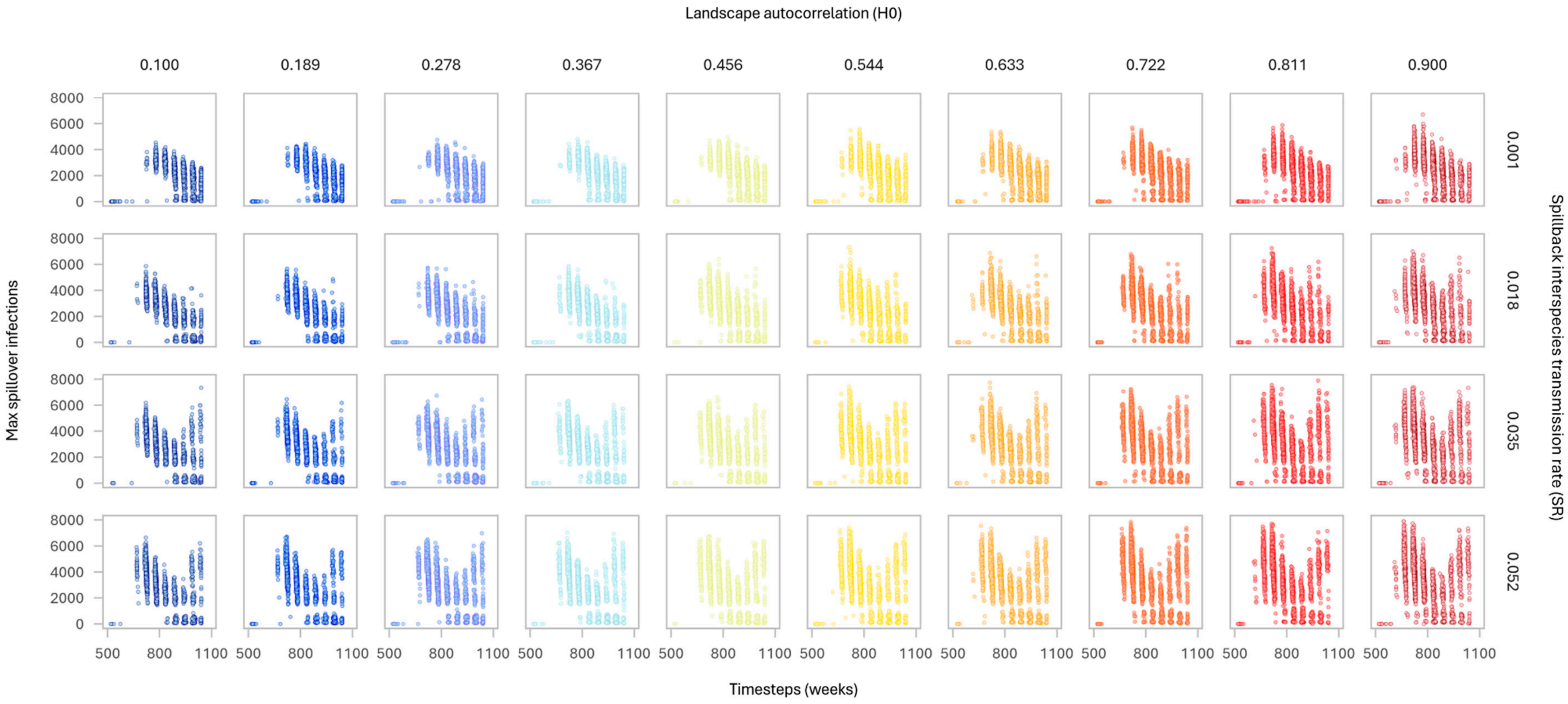

Figure S13. Timesteps (in weeks) at which the maximum number of spillover cases occurs, plotted against landscape autocorrelation (H_0) and spillback interspecies transmission rate (SR). Each dot represents a replicate simulation.

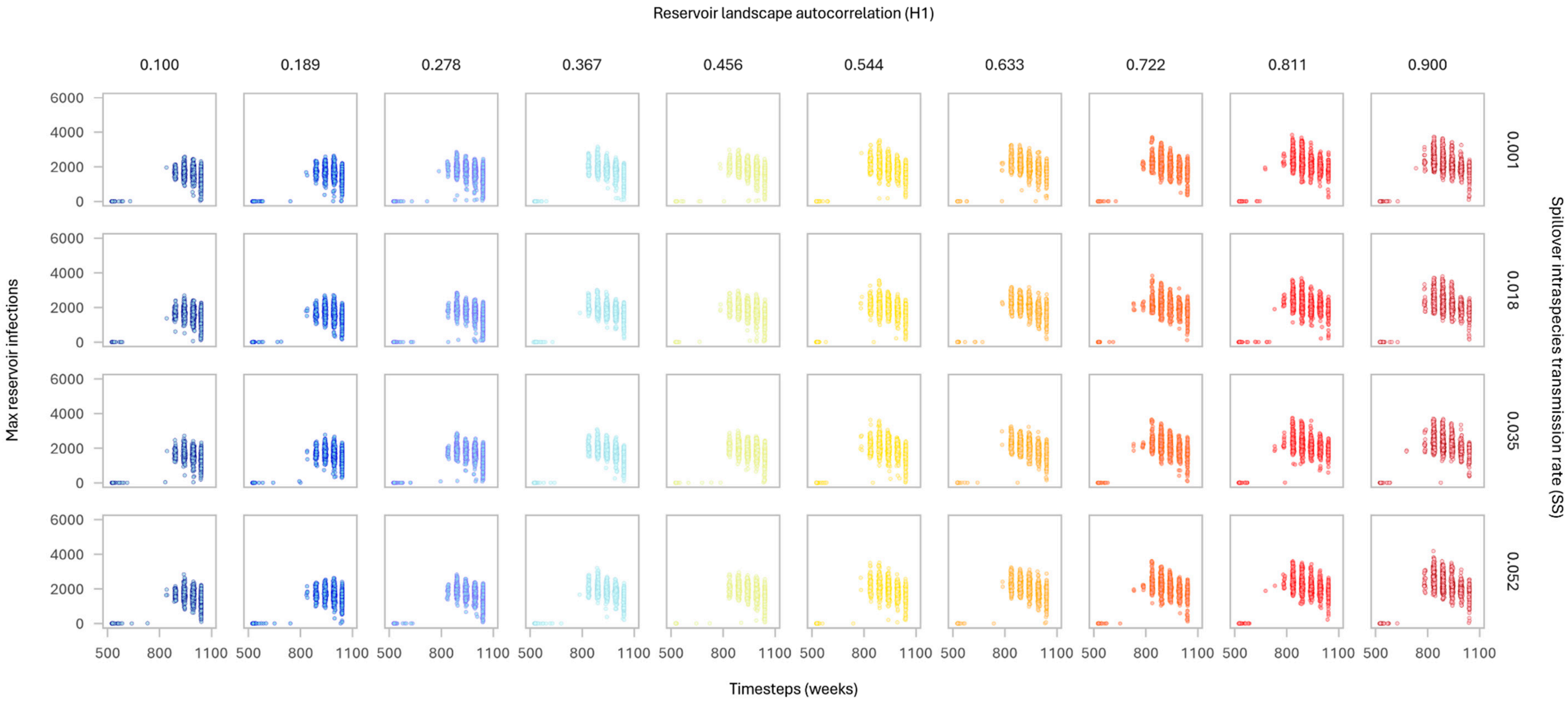

Figure S14. Timesteps (in weeks) at which the maximum number of reservoir cases occurs, plotted against reservoir landscape autocorrelation (H_1) and spillover intraspecies transmission rate (SS). Each dot represents a replicate simulation.

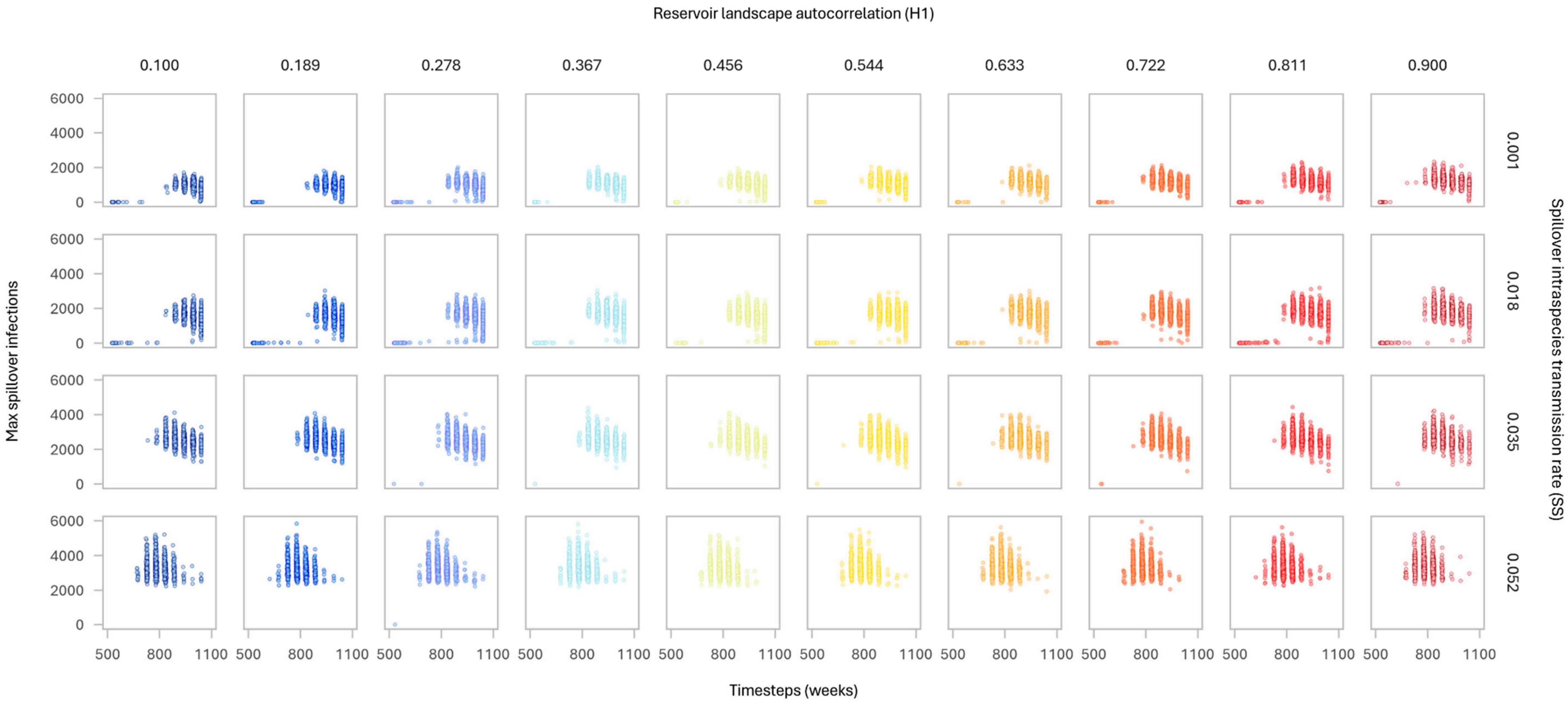

Figure S15. Timesteps (in weeks) at which the maximum number of spillover cases occurs, plotted against reservoir landscape autocorrelation (H_1) and spillover intraspecies transmission rate (SS). Each dot represents a replicate simulation.

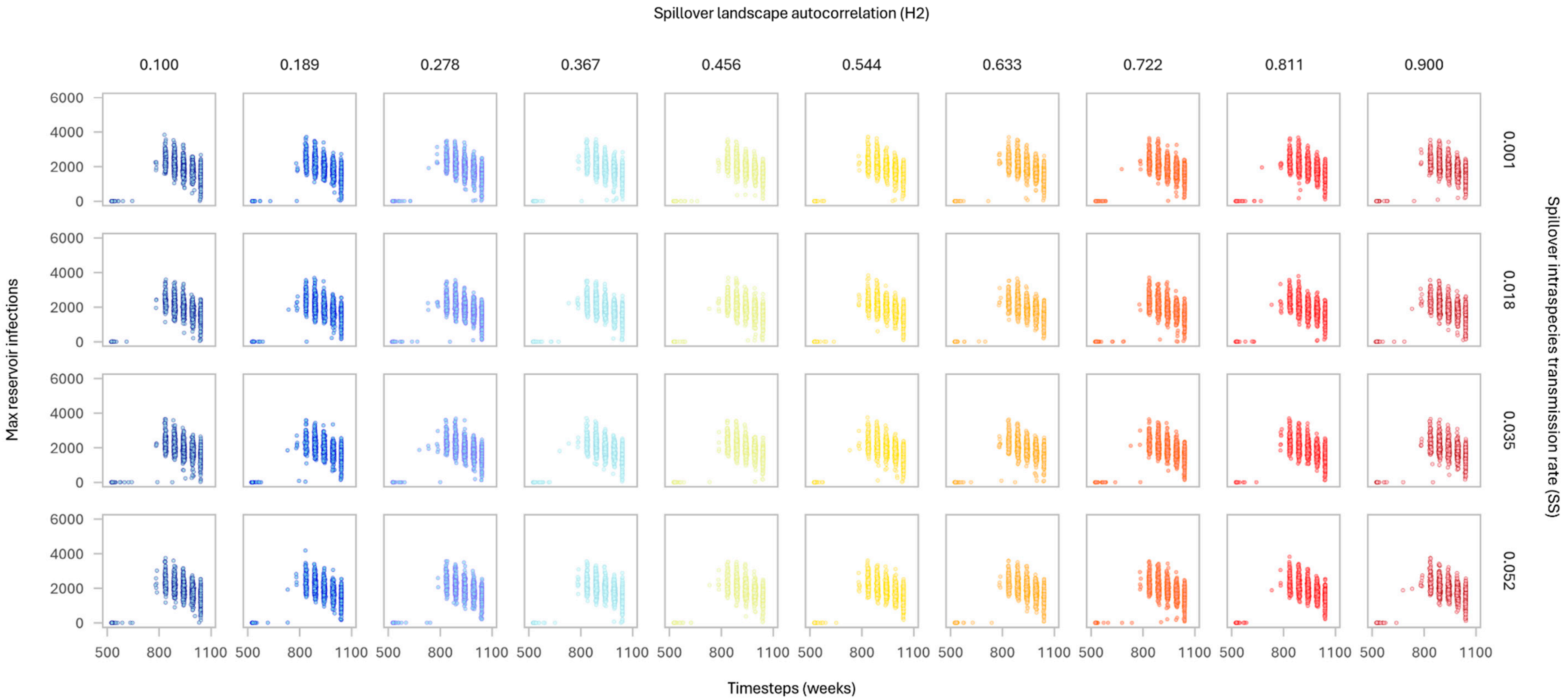

Figure S16. Timesteps (in weeks) at which the maximum number of reservoir cases occurs, plotted against spillover landscape autocorrelation (H_2) and spillover intraspecies transmission rate (SS). Each dot represents a replicate simulation.

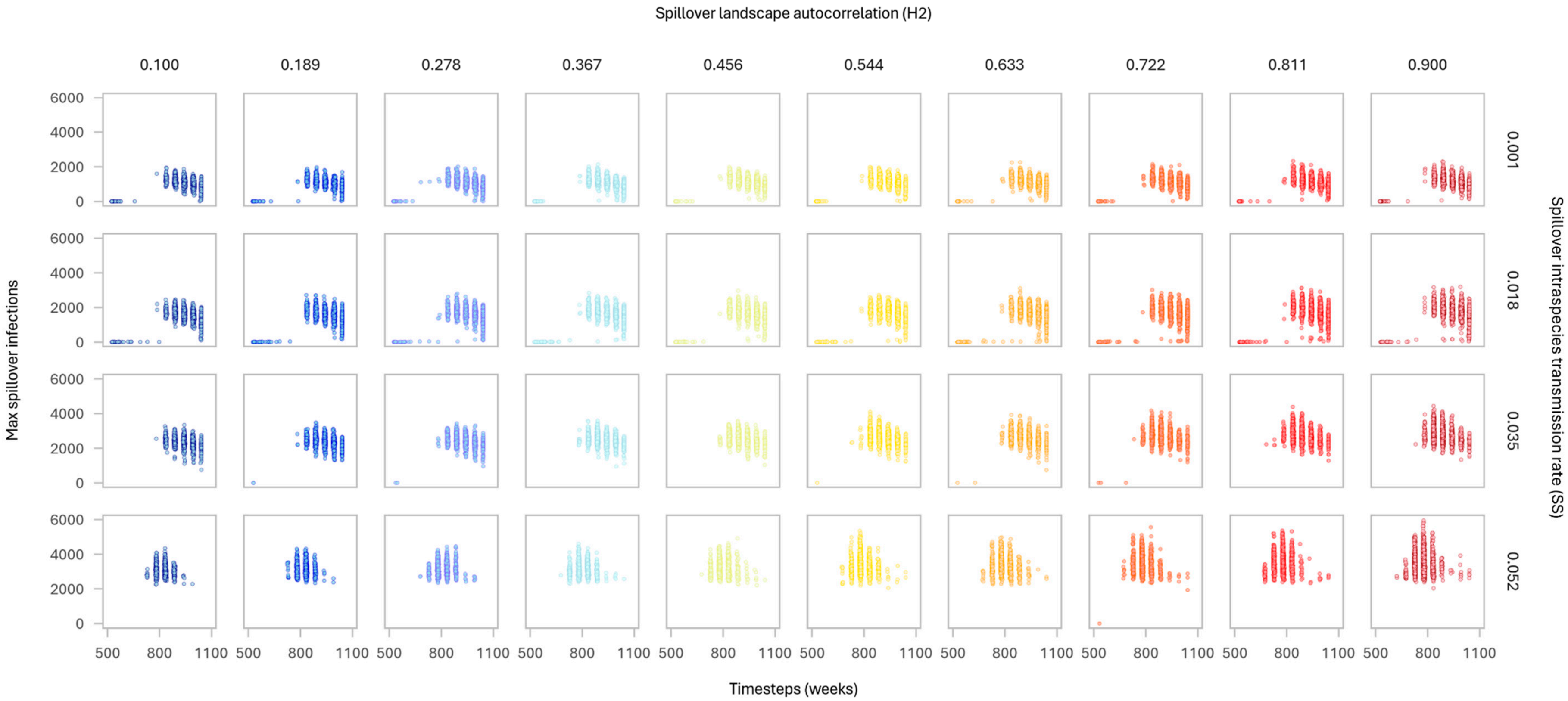

Figure S17. Timesteps (in weeks) at which the maximum number of spillover cases occurs, plotted against spillover landscape autocorrelation (H_2) and spillover intraspecies transmission rate (SS). Each dot represents a replicate simulation.
